# Could the tumor-associated microbiota be the new multi-faceted player in the tumor microenvironment?

**DOI:** 10.3389/fonc.2023.1185163

**Published:** 2023-05-23

**Authors:** Anne-Gaëlle Goubet

**Affiliations:** ^1^ Department of Pathology and Immunology, Faculty of Medicine, University of Geneva, Geneva, Switzerland; ^2^ AGORA Cancer Research Center, Lausanne, Switzerland; ^3^ Swiss Cancer Center Léman, Lausanne, Switzerland

**Keywords:** microbiome, microbiota, cancer, tumor, tumor microenvironment, tumorassociated microbiota (6)

## Abstract

Microorganisms have been identified in tumor specimens for over a century. It is only in recent years that tumor-associated microbiota has become a rapidly expanding field. Assessment techniques encompass methods at the frontiers of molecular biology, microbiology, and histology, requiring a transdisciplinary process to carefully decipher this new component of the tumor microenvironment. Due to the low biomass, the study of tumor-associated microbiota poses technical, analytical, biological, and clinical challenges and must be approached as a whole. To date, several studies have begun to shed light on the composition, functions, and clinical relevance of the tumor-associated microbiota. This new piece of the tumor microenvironment puzzle could potentially change the way we think about and treat patients with cancer.

## Introduction

Human body is composed of 30 x 10^12 eukaryote cells governing multiple and coordinated functions (1). Although endowed with very diverse and specific functions, the origin and development of eukaryotic cells is one of the most enigmatic processes of evolution. A crucial event in this process was the emergence of the mitochondria, the energy-generating organelles specific to eukaryotic cells. These organelles are thought to have begun to form when a bacterial cell related to the alpha-proteobacteria began living inside an archaeal host cell, of the phylum Lokiarcheaeota (2, 3). This event resulted in one organism living inside another (endosymbiosis) and was beneficial to both protagonists, who aligned their interests and evolved in synergy, thus becoming evolutionarily stable (4). This prolonged partnership and associated coevolution have thus led to the formation of the eukaryotic cell as we know it today which constitutes the main element of animals, plants, fungi, and protists. This symbiotic event occurred billion years ago (5, 6) and this is the first of a long series.

All plants and animals are home to symbiotic microorganisms whose interactions on the host can be neutral (commensalism), harmful (parasitism) or have beneficial effects (mutualism). These interactions are an integral part of coevolution and dynamically change from one to the other along a continuum depending on exterior and host factors (7). In the human body, viruses, bacteria, archaea, nanoarchaea, and eukaryotic microbes (fungi, protozoan, and parasitic helminths) constitute these symbiotic microorganisms and together form the microbiota. The number of microbial cells is roughly equal to the number of human cells (about 40 x 10^12 microbial cells) but their gene count exceeds the human genome’s gene count by ~100-fold (1). Host-microorganism interactions occur daily and take place in the skin and genital, respiratory (from the nose to the lung) and gastrointestinal (GI) tracts, which contains, by far, the greatest density and diversity of microorganisms. Long considered to be sterile, microorganisms have also been detected in the urine outside of any clinicopathological situation (8).

In human, ~97% of total microbial cells are bacteria residing in colon and ~2-3% are extracolonic bacteria residing in other body sites (gut, skin, lungs, etc). Therefore, bacteria largely dominate the human microbiome, where only ~0.1-1% are archaea, eukaryotic microbes and viruses in the GI tract (1, 9). The microbiota symbiotically contributes to several functions such as trophism, metabolism, barrier function, immunological processes, and signaling to virtually all organs of the body (10).

Beyond the beneficial homeostatic roles, a growing body of evidence suggests that microbes also influence states of health and disease including cancer. Indeed, the International Agency for Research on Cancer classifies 11 microbial agents (7 viruses, 3 parasites, and 1 bacterium) as group 1 human carcinogens and infection-induced cancer accounts for approximately 13% of the global burden of all human cancers (11, 12). Furthermore, it is now clear that microorganisms in the gut microbiota may also contribute to the carcinogenesis and prognosis of patients with cancer at the systemic level through mechanisms involving microbiota-derived metabolites, genotoxins and inflammation (13–15). Conversely, tumorigenesis can result from the contraction of anti-tumorigenic bacteria that release anti-proliferative metabolites. An endogenous strain of the mouse microbiota (*Faecalibaculum rodentium* isolate PB1) and its human counterpart *Holdemanella biformis* belonging to the Erysipelotrichaceae family, which disappear during the early phases of colorectal tumorigenesis, produce short-fatty acids that block tumor cell proliferation by reducing activation of nuclear factor of activated T cells, cytoplasmic 3 (NFATc3) and calcineurin (16). Then, there is growing evidence that micro-organisms, particularly bacteria and fungi residing in the gut, influence the response to chemotherapy, radiotherapy and immunotherapy with immune checkpoint blockers (14, 17). Because of its richness, diversity, and the ease of access to samples, the bacterial microbiome of the gut is at the center of research in this area.

An oxygen gradient along the longitudinal axis of the gut lumen, from ~6% O2 in the duodenal lumen to ~0.6% O2 in the colonic lumen, is generated and maintained by the host (18). Host factors and diet, by setting oxygen and nitrate availability, regulate and direct microbial growth that governs the composition and function of the gut microbiota. Thus, oxygen and nitrate deprivation in the lumen of the large intestine promotes the growth of obligate anaerobic bacteria. In tumors, oxygen levels range from 0.3% to 2.2%, which is 7-fold lower than the normal oxygen level measured in the corresponding normal tissue (19). In addition, tumors typically have aberrant, leaky, and irregular vasculature and create a gradient of small molecules (aspartate, ribose etc.) that act as chemoattractants for bacteria (20, 21). Tumors consist of an asynchronous and heterogeneous aggregate of malignant cells and host cells, such as immune and stromal cells entangled in an extracellular matrix. Tumor-associated immune cells, fibroblasts and endothelial cells associated are each phenotypically and functionally diverse; therefore, some can promote tumor progression whereas others can exhibit antitumor activity. However, when viewed as a whole, a tumor is an immunosuppressed structure where bacteria might more easily replicate without the clearance mechanisms of macrophages and neutrophils, which normally serve to eliminate them. Altogether, these characteristic (deoxygenation, chemotaxis, chaotic vasculature, immunosuppression) proper to the tumor microenvironment (TME) provide a favorable and attractive niche for microbial growth. Microorganisms have been identified in patient tumors for more than a century, although the magnitude of these microorganisms, their role and their interaction with components of the TME have been incompletely appreciated so far, mainly due to technological limitations.

After a brief description of the composition of the tumor-associated microbiota, the potential origin, assessment techniques and clinical relevance of this potential new player in the TME will be discussed.

## Composition and origin of the tumor microbiota

Tumor microenvironment includes microbes that reside in the tumor. To date, bacteria and fungi have been identified in several types of cancer and are significantly enriched when compared to adjacent normal tissue in several types of cancer (22–26). These microorganisms are present in the extracellular milieu, adhering or not to cells, and intracellularly. The intracellular localization concerns tumor and immune cells, especially macrophages. However, it is unclear whether intracellular bacteria penetrate these latter cells in an active or passive process. In primary tumors, the proportions of bacterial reads were significantly higher than fungal reads and the two were positively correlated together (24). Comparison of the beta-diversity between all pairs of tumor samples (n=528) within a given tumor type and across different tumor types revealed that microbiomes from tumor of the same type tend to be more similar to each other than they are to microbiomes from other tumor types (22). Species belonging to the Proteobacteria phyla account for most of the bacterial sequences detected (22, 26–29). Species belonging to Actinobacteria, Bacteroidetes and Firmicutes phyla are detected more inconsistently, appearing to vary between tumor types. In their Review, Sepich-Poore et al., by assuming tissue homogeneity estimated an average pan-cancer percent bacteria composition at 0.68% (bootstrapped 95% confidence interval of mean: [0.52%, 0.87%], 1000 iterations), with individual tumors ranging from absence to nearly 70% bacterial by cell count, corresponding to ~10^5^ to 10^6^ bacteria per palpable 1-cm^3^ (14). However, this estimation needs to be reviewed as bacterial distribution within tumor samples seems not to be homogeneous (30). Indeed, a study of cancers in the extremities of the GI tract - oral squamous cell carcinoma (OSCC) and colorectal cancer (CRC) - showed a heterogeneous distribution of tumor-associated bacteria in a subset of patients and a heterogeneous composition among these patients (30). Interestingly, other studies have shown that the presence of bacteria and fungi associated with tumors is not limited to malignant tumors of the digestive system, but also concerns tumors outside the digestive system such as bone and brain tumors for example (22–25, 31–35). These unexpected localizations raise the question of the origin of these microorganisms. It is worth mentioning here that the precise origin of intratumoral bacteria remains unknown; therefore, the main mechanisms proposed to explain the presence of intratumoral bacteria in different types of cancers are discussed in the following paragraph. As mentioned in the introduction, 11 microorganisms are causative agents of cancer. In this defined situation, these specific microbes can be found in tumors whose development they are responsible for following the acquisition of pathogenic strains earlier in life (36). Although not accepted as a causative agent of bladder cancer, uropathogenic *Escherichia coli* (UPEC) has been associated with the development of bladder cancer (37) and has been found in urothelial cancer cells and in tumor-associated immune cells in the bladder (35). Similarly, colibactin-producing pks+ *E. coli* has been associated with the development of CRC (38) and has been detected in primary and liver metastasis of CRC (39). Despite these specific cases in which a defined bacterium, fungi or virus might profoundly dominate the composition of the tumor-associated microbiota, the microbes present in tumors are diverse, suggesting more complex interactions. When a cancer develops near sites rich in microbiota, a direct colonization of the tumor can be established, as it could be the case for melanoma (skin) and non-small cell lung cancer (NSCLC) (lung). In the same logic, specific pathogenic fungi or bacteria residing in the duodenum, to which the pancreatic duct opens, could be a source of pancreatic ductal adenocarcinoma (PDAC)-associated bacteria due to retrograde bacterial and fungal migration from the duodenum to the pancreas (40, 41). Indeed, using mouse models, it has been shown that the gut microbiota modulates the PDAC tumor microbiome landscape and that fecal microbial transplants can modulate tumor immunosuppression and growth (27, 41). For other location, the most plausible origin is a translocation from a different anatomical compartment, such as the gut or the skin, to the tumor through the blood or the lymph. Microbial reads have been detected in blood from patients with or without cancer (25). However, more work is still needed to determine whether those detected nucleic acids come from live microorganisms, host cells, or lysed bacteria. Furthermore, it has been becoming clear that, under homeostatic condition, bacteria can penetrate the intestinal mucosa and skin, diffuse to the draining lymph node, and disseminate systemically under homeostatic conditions exerting protective rather than pathogenic effects (42–46). This phenomenon is amplified in pathological conditions that compromise the integrity of the epithelium or vessel permeability, such as colitis, atopic dermatitis, and treatments that alter the gut barrier, such as cyclophosphamide (43, 47). Malignant processes can also increase intestinal permeability by causing atrophy of the ileal mucosa (48). In parallel, bacterial translocation is emerging as a relevant microbial function for several immune-mediated diseases (49, 50). However, the key factors that dictate bacterial translocation capacity remain unclear and may involve genetic and environmental factors. One class of microbes, called pathobionts, has the ability to switch from neutral, harmless symbionts to harmful agents. This context-dependent transition is a stochastic event that gives pathobionts the ability to cause disease in a wide range of forms, from minor infections to more serious chronic or invasive diseases. Because billions of *de novo* mutations are generated daily within the gut microbiome and allow for commensal diversification and adaptation, evolution within the host has been shown to drive stochasticity in the development and progression of microbiota-induced diseases (51). Furthermore, pathobionts are well adapted to proliferate beyond their normal niche (7) thus potentiating their ability to readily colonize tumors. Whether this occurs in the context of tumors remains to be defined.

Despite its low biomass, its heterogeneity and the uncertainties surrounding its origin, recent technological advances have allowed us to better understand this new ecosystem.

## Technical approaches to explore the tumor microbiota

**Figure 1:**
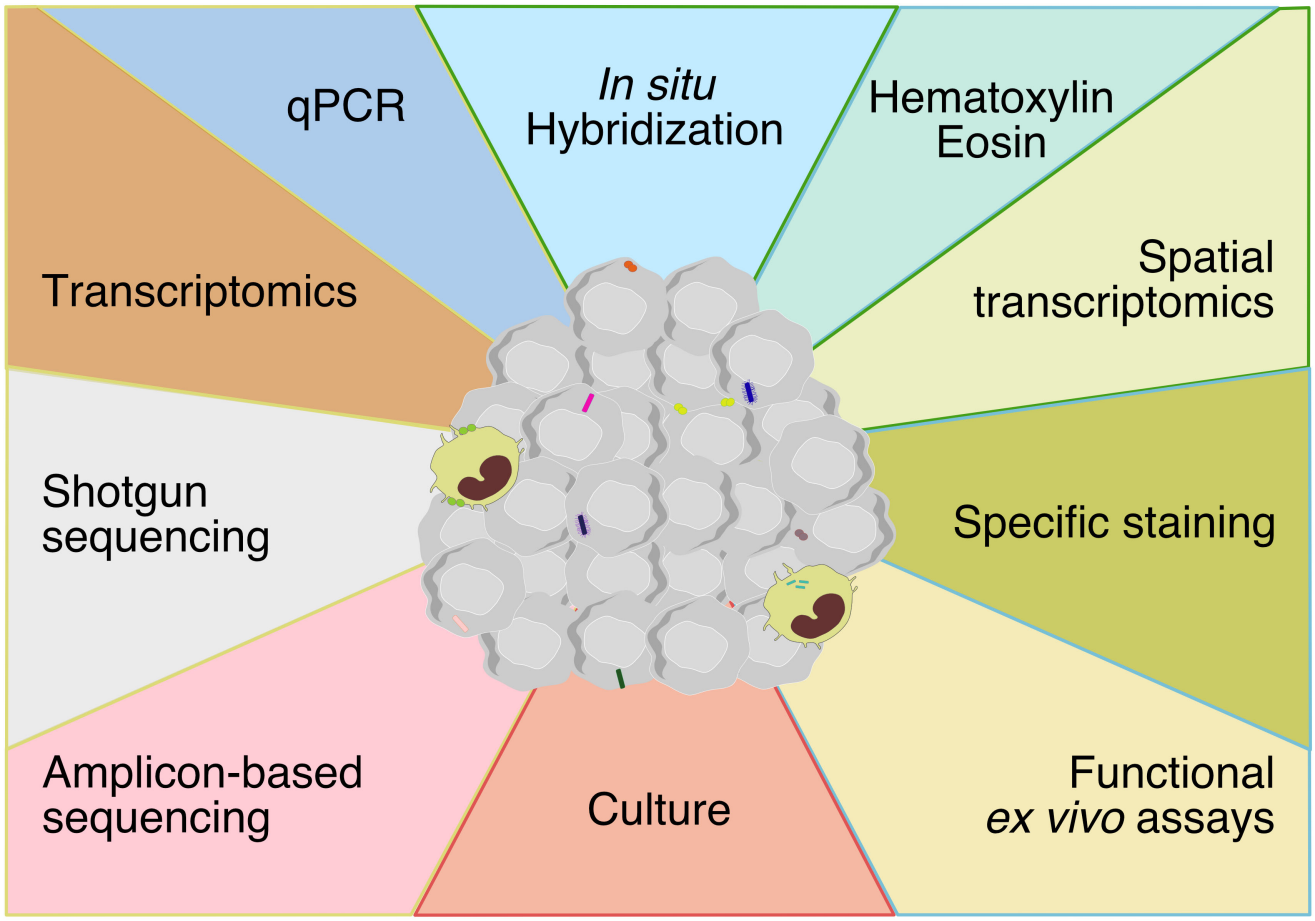
Techniques for assessing tumor-associated microbiota encompassing methods at the boundaries of molecular biology, microbiology and histology. The technical approaches to assess tumor-associated microbiota are methods based on molecular biology, microbiology and histology. Each color represents a method and each color line represent a discipline (yellow: molecular biology, red: microbiology, blue: histology, green: molecular biology-histology).

Recent technical advances have discredited the historical view that the inner organs are sterile in healthy individuals. Overall, technical approaches to assess tumor-associated microbiota are similar to conventional microbiology methods, based on culturomics and microbial culture- independent methods (**Figure 1**). These methods have converged to increase the sensibility and the specificity of detection. However, there are some limitations to consider.

### Molecular biology

This approach encompasses methods targeting either the bacterial DNA or bacterial RNA.

*DNA-based methods.* Historically, the sequence comparison of the small subunit ribosomal RNA (rRNA) gene from multiple different organisms enable to create the universal phylogenetic tree, thereby showing a scenario of three domains of life: Eubacteria, Archae, and the Eukaryota (52, 53). The 16s rRNA gene is present in almost all bacteria, its function has not changed over time, and its number of base pairs is sufficient for computational analysis, making it the most common genetic marker for studying bacterial phylogeny and taxonomy (54, 55). These features provide multiple molecular applications, such as FISH, qPCR and sequencing methods. The 16S rRNA gene comprises ~1500 bp with nine variable regions interspersed throughout the highly conserved 16S sequence (56). This juxtaposition of conserved regions and variable regions has the advantage of detecting all the bacteria (conserved regions) while allowing profiling (variable regions) of these bacteria in a sample.

All bacteria can be detected and quantified by qPCR using universal primers targeting conserved regions, while targeted primers specific for regions shared between group of bacteria or species allow detection and quantification of specific genera or species (57). However, qPCR relies on known bacterial sequences and thus has little discoverability. Instead, 16s rRNA gene sequencing allows a global approach to characterize the composition of the tumor-associated bacteria. PCR amplification of the 16s rRNA gene region of interest prior to sequencing guarantees high sensitivity, but a crucial step remains the selection of primers for this amplification, on which the accuracy of this technique depends (58, 59). Indeed, multiples studies have shown different regions vary in their taxonomic utility due to a combination of primer bias, differential hypervariable region sequence length, and hypervariable sequence uniqueness across bacterial taxa (60–64). Primers targeting the V2, V3, V4 and V6-7 regions appear to provide more accurate results while primer targeting the V9 regions are the worst (60, 64). The libraries obtained are sequenced and the sequences are assigned a taxonomy using algorithms and bioinformatic tools are used for the analyses. Similarly, the presence of fungi can be studied using Internal Transcribed Spacer sequencing. Specific identification of archaea is more difficult due to an incomplete reference database and inadequate primers (65). rRNA gene techniques (qPCR and amplicon sequencing) represent a simple and cost-effective approach to profiling microorganisms, even those that are difficult or impossible to detect by culture methods. Therefore, these technics have been successfully used in several studies demonstrating the presence of bacteria within tumor. However, in tumor samples, the ratio of human to microbial DNA is overwhelming and off-target amplification of human DNA with this technique has been reported (66). This artifact could be overcome using a DNA extraction kit that allowed microbial DNA enrichment (67) or by optimizing qPCR protocol at several key steps. The latter can achieve a detection sensitivity of 5,000 bacteria per gram of tissue (34). Targeting sub-regions represents a historical compromise, due to technology restrictions (56). New strategies have been developed to increase the accuracy and coverage of bacterial 16S rRNA sequencing, including multiplexed sequencing that relied on co-amplifying multiple hypervariable regions followed by sequencing (27, 60, 64, 68) and high-throughput sequencing of the full gene (69, 70). Therefore, it has recently been shown that whole-gene sequencing (long-read sequencing) provides better taxonomic resolution than targeted sequencing of variable regions, allowing taxonomic resolution to be extended to the specie and strain levels, especially. However, sequencing of the full-length 16s rRNA gene has not yet been tested on tumor samples.

Over the past decades, next generation sequencing (NGS) (mainly whole genome sequencing and whole transcriptome sequencing data) revolutionized our understanding of human diseases, including cancer (71, 72). Applied to the field of microbiology, this tool (called “shotgun metagenomic”) determines the DNA sequence of multiple bacterial genomes in a single sequence run, which provides, among other things, information on resistance and virulence, as well as information for typing (73). This advent tool enabled the Human Microbiome Project, which has published a wide range of data on the human microbiome (74–76). Comprehensive computational tools have been developed to identify nonhuman nucleic acids that may indicate candidate microbes (virus, fungi and bacteria) in high-throughput sequencing data from human cancer tissues such as PathSeq (77), CaPSID (78) and PathoScope 2.0 (79) and virMAP (80). As a result, a multitude of studies have been inspired by these approached and developed adapted pipeline to profile the microbiome from The Cancer Genome Atlas (TCGA) sequencing datasets (26, 81–86). This method involves sequencing random fragments of DNA. Therefore, in addition to host DNA in the sample that confounds microbial identification, several microbial reads are the results of contamination and that distinguishing contaminants from tissue processing and biologically relevant microbes remained a challenge for use of the human sequencing datasets. Thus, several strategies have been deployed to identify and mitigating contamination in TCGA sequencing data, through statistical model, through examination of batch effects and through careful consideration of all bacterial taxa present and their relative abundance (Poore et al., 2020; Robinson et al., 2017; Dohlman et al., 2021). Recently, Poore et al., re-examined treatment-naïve whole genome and whole transcriptome sequencing studies (n=18,116 samples) from 33 cancer types in TCGA for microbial reads (25). They found unique microbial signatures in tissue and blood within and between most major cancer types demonstrating the potential of blood microbial DNA to diagnose cancer. In parallel, Dohlman et al., presented The Cancer Microbiome Atlas, a collection of curated, decontaminated microbial compositions of oropharyngeal, esophageal, gastrointestinal, and colorectal tissues (87). This led to the discovery of prognostic species and blood signatures of mucosal barrier injuries, a common feature of colorectal cancer and other chronic inflammatory conditions.

Multiple elements must be considered to ensure an unbiased and comprehensive approach. The large inter-individual heterogeneity in the composition of the gut microbiota is due to human lifestyle and physiological variables (88–90). Therefore, we cannot exclude an effect of antibiotic, non-antibiotic (human-targeted) drugs, frequency of alcohol consumption, bowel movement quality, and dietary intake on the tumor-associated microbiota as well. Ideally, studies should be designed to collect all potential confounding factors and match cases and controls equally, taking these confounders into account, to decrease the risk of false positives results and reduce spurious microbial associations with cancer. Alternatively and depending on the aim of the study, by including matched samples (tumor and adjacent non tumor tissue), each patient can serve as his or her own control thus reducing interaction and confounding factors (26, 28). Archived formalin-fixed paraffin-embedded (FFPE) tissues are a source of clinical and molecular information that have been largely employed to explore tumor-associated microbiota in the DNA-based techniques. However, this approach presents several limitations: i) FFPE tissue section may not be representative of the whole tumor, ii) fixation steps could alter the quantity and the quality of information obtainable from FFPE, iii) sample degradation over time (91). The feasibility of using FFPE samples for NGS analysis has been questioned because the formalin fixation process can cause disruptions in the nucleic acids such as fragmentation and mutations. For this reason, some studies used a commercial kit designed for NGS applications, that enzymatically removes artificial C > T mutations due to cytosine deamination, which are artifacts caused by formalin fixation and aging. Therefore, these technical limitations must be considered before studying tumor-associated microbiota.

In addition to these pre-analytical limitations, there are multiple elements to consider when analyzing these data sets. Regarding shotgun metagenomic studies, metagenome assembly is a critical step to guarantee very high-quality genomes for accurate analyses (92): it is important to ensure that DNA sequences from metagenomes are assembled into nearly pure and complete representations of the individual strains that make up these communities allowing an accurate identification of the species and of their functions. Methods for obtaining metagenome-assembled genome have varying degrees of success based on the community composition, the library preparation and sequencing technologies, and the computational algorithms used for assembly (93, 94). More generally, datasets collected by high-throughput sequencing of 16S rRNA gene amplimers, metagenomes or meta transcriptomes are compositional, as the instrument imposes an arbitrary total number of reads that cannot be related to the absolute number of molecules in the input sample (95). Therefore, the results are relatively rather than absolutely quantitative, a notion that investigators should keep in mind when analyzing dataset by applying compositionally-appropriate tools to avoid misleading results (92). Finally, microbiota-related studies are increasing and allow for cross-cohort exploration for which sophistical tools are needed to exploit all the information in these biological datasets, taking into account the peculiarities of microbiome data, namely compositionality, heterogeneity, and sparse nature of these datasets (96, 97). In this regard, machine-learning provides new insights into the development of models that can be used to predict outputs. For instance, gut microbiome-based machine learning models was successful to predict the response to PD-1 blockade (98, 99).

*RNA-based methods*. While NGS has contributed to the current revival of molecular microbiology, single-cell transcriptomics has the potential to go beyond bulk methods and provide insight into expression differences between individual cells within a community (100). This latter method has become an essential tool for characterizing gene expression in eukaryote (101). Bacterial characteristics including a tough cell wall (covering the cellular membrane) hard to lyse, a low mRNA content (about 2 orders of magnitude lower than that of eukaryote cells), a lack of posttranscriptional modifications including the addition of the poly(A) tail, have delayed the implementation of single-cell genomics on bacterial cells. These different characteristics between eukaryote and prokaryote cells precluded a simple application of standard eukaryote protocols and require an adaptation of the methods such as microbial split-pool ligation transcriptomics (microSPLiT) (102). This approach was applied to free bacteria cells and uncovered a wide range of developmental and metabolic gene expression programs. In parallel, the development of the probe-independent RNA sequencing approach has begun to revolutionize transcriptomics allowing the emergence of dual RNA-seq, in which gene expression changes in both the pathogen and the host are analyzed simultaneously (103). Studies undertaking dual RNA-seq on *in vivo* samples have been performed on total tissues rich in extracellular bacteria (104–107) and on *Mycobacterium tuberculosis*-infected, ontogenetically distinct macrophage lineages isolated directly from murine lungs (108). In parallel, *in vitro* studies have led to the development of a single-cell dual RNA sequencing method (scDual-Seq) simultaneously capturing host and pathogen transcriptomes to track host and pathogen transcriptomes over the course of an infection (109, 110). Russell and colleagues developed an adapted approach to associate bacterial and host cell phenotype at the single cell level to simultaneously acquire the host transcriptome, surface marker expression, and bacterial phenotype for each infected cell *in vivo* (111). In parallel, two recent studies developed an analytical approach using the small-RNA sequencing (miRNA-seq) (112, 113). Compared to RNA-seq, miRNA-seq which is processed without poly-A filtering could have a chance to identify bacteria not found in RNA-seq. In addition, SAHMI, a computational resource for systematically retrieving and denoising microbial signals in human clinical tissues and assessing host-microbiome interactions at single-cell resolution, was developed, and applied to publicly available scRNA-seq datasets generated from known infection and PDAC samples (114, 115). While the authors did not detect any fungal or viral signals, they showed that bacteria were predominantly paired with tumor cells and that this status exhibited activated T cells that are transcriptionally more similar to T cells present in an infectious context than in other tumor types. Finally, Bullman and colleagues developed an alternative method for single-cell RNA sequencing by introducing a primer that targets a conserved region of bacterial 16S rRNA, facilitating the generation of cDNA libraries with bacterial transcripts from bacteria-associated human cells, which they named INVADEseq (invasion-adhesion-directed expression sequencing). Applying this method to patient fresh tumor samples, they identified cell-associated bacteria and the host cells with which they interact, while uncovering alterations in transcriptional pathways involved in inflammation, metastasis, cell dormancy, and DNA repair.

Therefore, single-cell transcriptomics in prokaryotes offers the possibility to dissect the host-microbe interaction *in vivo*, which is crucial for understanding the molecular mechanisms that govern the establishment, regulation, and role of the tumor-associated microbiota.

### Histology

While hematoxylin-eosin staining can contribute to the identification of microbe-like structures (30, 35, 116), basic microbiological stains, such as Gram or Grocott-Gomori’s methenamine silver stains, can contribute to a general detection of microbes in tissue samples (117), though sensitivity and specificity of these methods are low for characterization of tumor microbiome. In addition, the use of antibodies directed against microbe-associated particles, such as lipoteichoic acid for gram-positive bacteria, lipopolysaccharide for gram-negative bacteria, can be helpful for characterization of tumor microbiome, although positivity seems to represent mostly intracellularly processed or internalized particles, not necessarily a living microorganism (22, 27, 35). Probes binding regions of the 16s rRNA coding gene or its transcript are used to visualize bacteria on FFPE tissue samples by *in situ* hybridization (fluorescent (FISH) or RNA-scope). Combining universal bacterial 16s rDNA/rRNA-directed probe with genra-specific probe, this approach has been used to report an association of *Fusobacterium* with the colonic mucosa of colorectal carcinoma (85) and *E. coli* with bladder cancer (35). In parallel, RNA *in situ* hybridization imaging applied to OSCC and CRC specimens revealed the heterogeneous spatial distribution of bacterial communities with positive areas while other areas were devoid of signal. Using probes specific for EBV, HBV or HCV, the same approach has been applied to determine the role of virus in several cancer types. Because no staining method can detect all fungi in tissues, an integration of four staining methods with varying levels of sensitivity and specificity was used to describe the tumor mycobiome including: a fungal cell wall-specific anti-β-glucan antibody, an anti-*Aspergillus* antibody that also binds several additional fungal species and probes against three conserved fungal 28S rRNA sequences with selective sensitivity for yeast over hyphal morphologies due to lower hyphae probe penetration (24). Combining these methods with immune and non-immune targets, histological assays present the advantage to assess the spatial distribution of microbe in tumor tissues.

Moreover, confocal microscopy and electron microscopy could suggest whether the bacteria reside intracellularly or not (85, 118). For example, confocal imaging showed single cells from a tumor of a OSCC patient containing cell-adherent and intracellular bacteria (30). Although extremely useful to visualize and characterize the location of microbes, the techniques require the use of fresh samples to obtain good quality micrographs.

By combining high-plex profiling at the protein and RNA level, spatial transcriptomic assays have recently emerged to map tumor regions with cell type-specific genomic expression (119). Using a targeted approach through the GeoMx digital spatial profiling platform in combination with RNAscope for *F. nucleatum*, the expression profile of 77 proteins from either immune or epithelial compartments of OSCC and CRC showed that bacteria reside in immune microniches enriched for CD66b+, ARG1, CTLA4, PD-1 (30). However, the main limitation of this latter platform is the inability to provide single-cell resolution, thus limiting the understanding of cell-cell interactions and specific host-microbial interactions. Recently, CosMx spatial molecular imager (Nanostring technology) has emerged as the first high-plex *in situ* analysis platform to provide spatial multiomics with FFPE and fresh frozen tissue samples at cellular and subcellular resolution. This new technique will likely soon provide a better understanding of how tumor-bacteria associations affect TME function and composition.

While molecular biology and histological approaches provide unique tools to characterize the composition and localization of the tumor microbiota, it is hard to define the presence of living microorganism. Among available technique, the identification of signals for microbe-specific RNA favors the presence of living microorganisms, thus, being preferred to further characterize the host-pathobiont relationship within the TME. Flow cytometry kits (for free microbes) and functional ex-vivo assays, such as labeled D-Alanin uptake by tumor and/or immune cells, or tissue culture are the best tools to determine the viability of these potential pathobionts (22, 120, 121). However, it is worth to note that some bacteria might be viable but nonculturable (VBNC) or resting/non-proliferating bacteria, which could also result in negative results for these methods. Therefore, only large-scale microbial culture can isolate and certify viability with certainty.

### Culture

Each microorganism has its own culture requirements. For example, the majority of viruses and some intracellular bacteria are grown in cell culture systems, while atmospheric conditions are important for growing bacteria for which some species are killed by oxygen, while others need it to grow. In addition, many bacterial species could enter in a specific state called the VBNC state (122) under specific conditions. VBNC cells are characterized by a loss of “culturability” on routine agar, which impairs their detection by conventional plate count techniques. Altogether, this challenges the study of viable cells in tumor samples.

Consisting of multiple culture conditions combined with the identification of bacteria, the culturomic approach enables the culture of new microorganisms (123). Culturomics was initially developed to enable the growth of fastidious bacteria from the human gut by establishing a method offering multiple culture conditions followed by identification using mass spectrometry. Indeed, at that time, it was commonly accepted that 80% of the bacterial species found by molecular tools in the human gut were uncultured or even unculturable (76). In the first culturomic study, 212 culture conditions generated more than 30,000 colonies yielding to 340 bacterial species, including 31 new bacterial species and species belonging to rare phyla (124). The first step of culturomics is to divide the sample and diversify the sample into different culture conditions. These conditions are designed to suppress the culture of majority populations and to promote the growth of fastidious microorganisms present at lower concentrations. The improvement of culture media using blood and rumen fluid in blood culture bottles is one example to promote the growth of minority populations. Colonies are then isolated for identification by MALDI-TOF, which relies on the comparison of the protein mass spectra of the isolate with an upgradable database. If identification fails, the isolate is subjected to 16S rRNA sequencing. The discovery of new taxa is confirmed by genome sequencing, and taxonogenomics is used to formally describe the bacterium. All identification results are compared with a database that contains bacterial species recovered from humans. This technic has been applied to the gut and to the urinary microbiota, for instance that has increased the repertoire of bacterial species associated with humans thanks to the identification of new species (125). Applied to tumor-associated microbiota, this high-throughput microbial culture requires specific enhancement adapted to the requirements of the microbes present in these specific tissues. To date, this approach was used by Nejman et al. to show the presence of live bacteria within 5 breast tumor samples.

Isolating individual bacteria from complex microbial ecosystems is labor intensive, difficult to scale up and relies on a random colony selection method; machine learning has the potential to enhance this framework (126). Indeed, this bacterial culture remains essential as it has the main advantage of providing live and pure isolates of tissue-associated microbes, allowing for subsequent analysis and manipulation.

In general, standard microbiology methods developed for the analysis of specimens with a high bacterial load (e.g., stool) must be modified when the bacterial load is low (e.g., tumor), as background contamination derived from laboratory reagents although considered sterile or from the environment may dominate and distort observations. Overall, and as recently advised, the presence of microorganisms identified by sequencing should be verified using a different technique such as culture, a second sequencing technique with higher resolution, and/or species-specific qPCR or FISH using high magnification to visualize the size and morphology of individual microbial cells (127). Then, the authenticity of such signals should be examined taking into account the physiology of the human body.

## Role

Applying these different approaches to fresh and stored tumor samples, multiples studies highlighted the presence of bacteria and fungi within the tumors while showing association with clinical outcome (22–24, 27, 31, 33). Overall, tumor cells and macrophages are the main cellular host of these microbial inhabitants. Because macrophages are a professional cell type for microbe detection and phagocytosis, it is difficult to distinguish whether microbes are actively invading them or have been phagocytosed by these cells. Very little is known about the effects of these microbes on tumor-associated macrophages. Macrophages with bacteria engulfed showed significantly increased expression levels of genes that are involved in the inflammatory response through activation of TNF, INFγ and IFNα, and genes that are involved in the production of interleukins through the JAK–STAT signalling pathway, such as *IL1B*, *IL6* and *IL10* compared to those without bacteria and these signatures were independent of bacteria genus (30). Altogether, these findings suggest that it may be a snapshot of an active innate immune response against tumor-associated bacteria rather than a permanent residence in tumor-associated macrophages as is the case in some chronic bacterial infections (such as tuberculosis). Consistent with this hypothesis, another study showed by culture that while the number of bacteria in tumor cells did not differ from the number of bacteria found in the entire tumor tissue in immunocompetent mice, there was a trend toward increased extracellular bacterial components in immunodeficient mice (34). However, further studies are needed to clearly state the role played by these microbes on macrophages and whether the bacteria have acquired resistance or escape capabilities to the phagolysosome. Furthermore, Bullman and colleagues surprisingly showed a single cluster of monocyte-derived macrophages in tumor samples from 7 OSCC patients (30), whereas usually this population becomes very heterogeneous in TME (128). An increase in the number of samples and/or an adaptation of the analytical steps could allow the identification of a subtype of macrophages preferably associated with the bacteria. In parallel, this study also showed that an aneuploid epithelial cell cluster consisting of cancer cells contained most of the bacterial transcripts, as compared to other euploid epithelial cell clusters. Compared to bacteria negative cells, Fusobacterium- or Treponema-associated single cells presented an upregulation of IFN and JAK–STAT signaling with an increased expression of metalloproteinases, including MMP9 and MMP3. These differences disappeared when considering general bacteria-positive cells suggesting taxa-specific epithelial cell interactions rather than a general bacteria-induced response. Therefore, to directly evaluate the interactions of a dominant member of the CRC and OSCC microbiota with epithelial cancer cells, the authors used a reductionist approach co-culturing CRC spheroid with *F. nucleatum* to show that *F. nucleatum* induced neutrophils swamming and promoted transcriptional changes facilitating the detachment and the migration to the surrounding environment of infected cells. These results suggest that intratumoral bacteria may promote tumor cell invasiveness. In parallel, using a spontaneous mouse model of breast cancer and different routes of antibiotic administration, another study showed that bacteria residing in tumor cells were essential for spontaneous breast cancer metastasis. By reorganizing the cytoskeleton and promoting resistance to mechanical stress, certain bacterial species such as *Streptococcus cuniculi*, *Staphylococcus xylosus*, or *Lactobacillus animalis*, were able to promote metastasis without affecting the tumor burden of the primary tumor (34). These events originated in the primary tumors, promoting the migration of tumor cells harboring the bacteria to the metastatic sites. Analysis of the microbiota composition of matched normal and tumor human breast tissue and lymph node metastases by 16s sequencing showed that tumor tissue samples contained significantly higher abundance of *Enterococcus* and *Streptococcus* than adjacent normal breast tissue samples. Interestingly, lymph node metastases were closely associated with the tumor microbiota, supporting the idea that microbes in metastases are inherited from the primary tumor. This study suggested that bacteria can, but only together with tumor cells, travel through circulation system and colonized in distal organs. However, another study demonstrated that bacteria disseminated to the liver and induced the premetastatic niche formation (39). In their study, the authors nicely demonstrated that specific CRC resident bacteria, such as *E. coli*, upregulated the expression of an endothelial marker, PV-1, which was associated with increased permeability of intestinal blood vessels and was found to be an independent marker of distant CRC recurrence (129, 130). This alteration of intestinal vessels facilitated translocation of bacteria to the liver where they promoted myeloid infiltration prior to the development of distant metastases. Interestingly, both events, the increase in PV-1 and the enrichment of innate immune cells in the liver, require specific bacteria and most likely specific genes expressed by these bacteria to occur. Unlike the two previous studies, this study showed how the bacteria themselves can promote metastasis by preparing the remote site for seeding of cancer cells without involving a direct effect on the cancer cells.

Bacteria extend the enzymatic repertoire of humans in general in a beneficial way such as in the intestine by participating in the digestion and assimilation of nutrients and vitamins, but they can also alter the efficacy of multiple chemotherapies (131–133). At the tumor site, deleterious enzymatic activity has been reported for bacteria in PDAC (40). Some of these bacteria possessing the long isoform of the bacterial enzyme cytidine deaminase (CDD-L), seen primarily in Gammaproteobacteria, were involved in the inactivation of gemcitabine, suggesting that intratumor bacteria might contribute to drug resistance. Consistent with this possibility, the authors found that 86/113 (76%) tested human PDAC were positive for bacteria, mainly Gammaproteobacteria. Similarly, some bacteria are able to deplete 5-FU, reducing the efficacy of the drug against human colorectal cancer tumor epithelial cells. This effect is probably not mediated by soluble factors secreted by the bacteria and appears to involve the *preT* and *preA* genes responsible for metabolizing uracil to dihydrouracil sharing homology with human dihydropyrimidine dehydrogenase which has been shown to detoxify 5-FU to dihydrofluorouracil (134).

In addition to these negative effects, protein fragments from bacteria that invade tumor cells can be presented on the surface of tumor cells and recognized by the immune system (68). Applying immunopeptidomics to 17 melanoma samples, Samuels and colleagues found that many peptides associated with HLA molecules were derived from 38 bacteria. At the same time, a subset of these peptides was presented by both antigen-presenting cells (APC) and tumor cells, indicating that the same peptide can both trigger an immune response through its presentation on APCs and be a target for an immune attack on tumor cells. Interestingly, some peptides were found in several metastases from the same patient, while others were found in samples from different patients. These results show that bacteria constitute a completely new source of tumor antigen. Two categories are classically described: tumor-associated and tumor-specific antigens. The former is expressed by both tumor cells and non-tumor cells. These antigens are shared among patients and are poorly recognized by the immune system. When recognized, this increases the risk of autoimmunity. Instead, the latter are expressed only by tumor cells and are therefore an ideal target for the immune system. But they are unique to each patient. Bacterial-derived antigens are therefore at the junction between these two types of antigens: they are shared by several patients, differ between non-tumor and tumor tissue, and are strongly recognized by the immune system. By avoiding therapeutic personalization, these new peptides/antigens could open the way to new immunotherapies against cancer. However, to date there was no direct proof of their clinical relevance. Our study showed that the UPEC-specific humoral immune response may have clinical significance in patients with locally advanced and metastatic bladder cancer treated with anti-PD-1 mAbs (35). *E. coli* could be detected in bladder cancer by a combination of techniques (PCR, fluorescent *in situ* hybridization, FISH). While all bacteria tested stimulated IFNg release from CD4+ memory T cells, E. coli was the best inducer of CXCL13 release. Interestingly, *E. coli*-specific CXCL13 release from CD4+ memory T cells was highest among samples from MIBC patients who responded to neoadjuvant pembrolizumab. In addition, serum IgG directed against invasive E. coli in the urothelium (but not against any other urinary commensal) was associated with favorable clinical responses in three independent retrospective cohorts. In parallel, exposure of fresh muscle-invasive bladder cancer immediately after cystectomy to an exogenous strain of UPEC triggered activation of intratumoral TFH and antibody-secreting cells, resulting in the release of CXCL13, CCL19, and CCL21, all of which are prototypical TLS-related chemokines. These results suggest the possibility of exploiting the local microbiota, in particular UPEC, as an adjuvant to PD-1/PD-L1 blockade.

## Conclusions and perspectives

The study of the tumor-associated microbiota is a growing field and is, currently broadening our understanding of its origins and functions. The techniques used to explore the tumor microbiota must be sufficiently sensitive and specific to allow adequate characterization. Current methods include microscopy, genomics-based techniques, and microbial cultures, which need to be combined to obtain an accurate description. Improvements in these techniques for specific application to low biomass hypoxic tissues containing intra-, extracellular, and adherent microbes, such as tumors, are likely to be made in the near future. In addition to including the necessary controls to avoid interpretation bias due to contaminants, it is necessary to develop genomic techniques inspired by microbiology to enrich the microbial genomes with respect to the human genome and to study both in parallel. Beyond these technical limitations, the search for tumor-associated microbiota must be part of a transdisciplinary approach including microbiological, immunological, and oncological concepts. It is becoming increasingly clear that the microbes present in tumors influence, for some, the development and, more globally, the prognosis of cancer. At the same time, these microbes constitute an extremely rich source of danger signals and of non-self-antigens making them stimulating agents and targets of the immune system that can potentially influence the response to immunotherapies. Finally, it seems sensible to assume that modulations of the gut microbiota may have an impact on the tumor microbiota, since the former is most likely the main source of the latter. While all classes of antibiotics alter the composition of the gut microbiota, the tumor microbiota may be more sensitive to antibiotics that diffuse intracellularly. We will probably learn in the coming years how to exploit and modulate this tumor microbiota to improve the management of patients with cancer.

## Definitions

Coevolution is the process by which two or more species evolve in tandem by exerting influences on each other.

Beta-diversity quantifies differences in the overall taxonomic composition between samples.

Pathobionts are commensal microbes (symbionts) that are harmless in a host with a functioning immune system and a “healthy microbiota”, but can proliferate and cause chronic inflammatory states under certain conditions, usually reflecting a dysfunctional immune state.

Culturomics is a high-throughput culture method that uses MALDI-TOF mass spectrometry to identify bacterial species.

## Author contributions

A-GG designed, drafted, and wrote the review.
